# South Asia's cardiometabolic burden: exposomal determinants of a health transition

**DOI:** 10.1016/j.lansea.2026.100881

**Published:** 2026-10-07

**Authors:** Yashendra Sethi, Anoop Misra

**Affiliations:** a Department of Medicine, Subharti Medical College, Swami Vivekanand Subharti University, Meerut, 250005, India; b Nexoria Institute of Cardiometabolic Excellence (NICE), Vigyaved Healthcare, Dehradun, 248001, India; c Fortis CDOC Centre of Excellence for Diabetes, Metabolic Diseases and Endocrinology, New Delhi, India; d National Diabetes Obesity and Cholesterol Foundation (N-DOC), New Delhi, India; e Diabetes Foundation India, New Delhi, India

South Asia is facing an escalating burden of obesity, type 2 diabetes (T2D), dyslipidemia, metabolic dysfunction–associated steatotic liver disease (MASLD), and premature atherosclerotic cardiovascular disease.^1^, ^2^, ^3^, ^4^ In 2023, high systolic blood pressure (SBP) accounted for nearly 138 million disability-adjusted life years (DALYs) and 6.27 million deaths across the Asia–Pacific region, while high body mass index, elevated LDL cholesterol, and T2D collectively contributed to more than 150 million DALYs. Since 1990, the cardiometabolic disease burden, measured by DALYs, has increased 1.7- to 3.7-fold.^3^ Importantly, South Asians develop cardiometabolic disease at younger ages and at substantially lower BMI than Western populations.^2^^,^^5^ Against this backdrop, we propose that South Asia may represent one of the world's largest ongoing natural experiments in exposome-mediated (mediated by the totality of environmental exposures) cardiometabolic disease, which cannot be explained by excess calorie consumption and physical activity alone. Within a single generation, South Asia has undergone metabolic transition, with rapid urbanisation, mechanisation, and dietary change superimposed on populations that experienced famine and undernutrition^1^^,^^2^ leading to adaptations favouring efficient energy storage and intra-abdominal visceral fat deposition. These adaptations now intersect with the exposome, with a different set of determinants, the cumulative burden of environmental exposures, including ultra-processed diets, endocrine-disrupting chemicals (EDCs), circadian disruption, climate stress, and stressful lifestyles (Fig. 1).

**Fig. 1:**
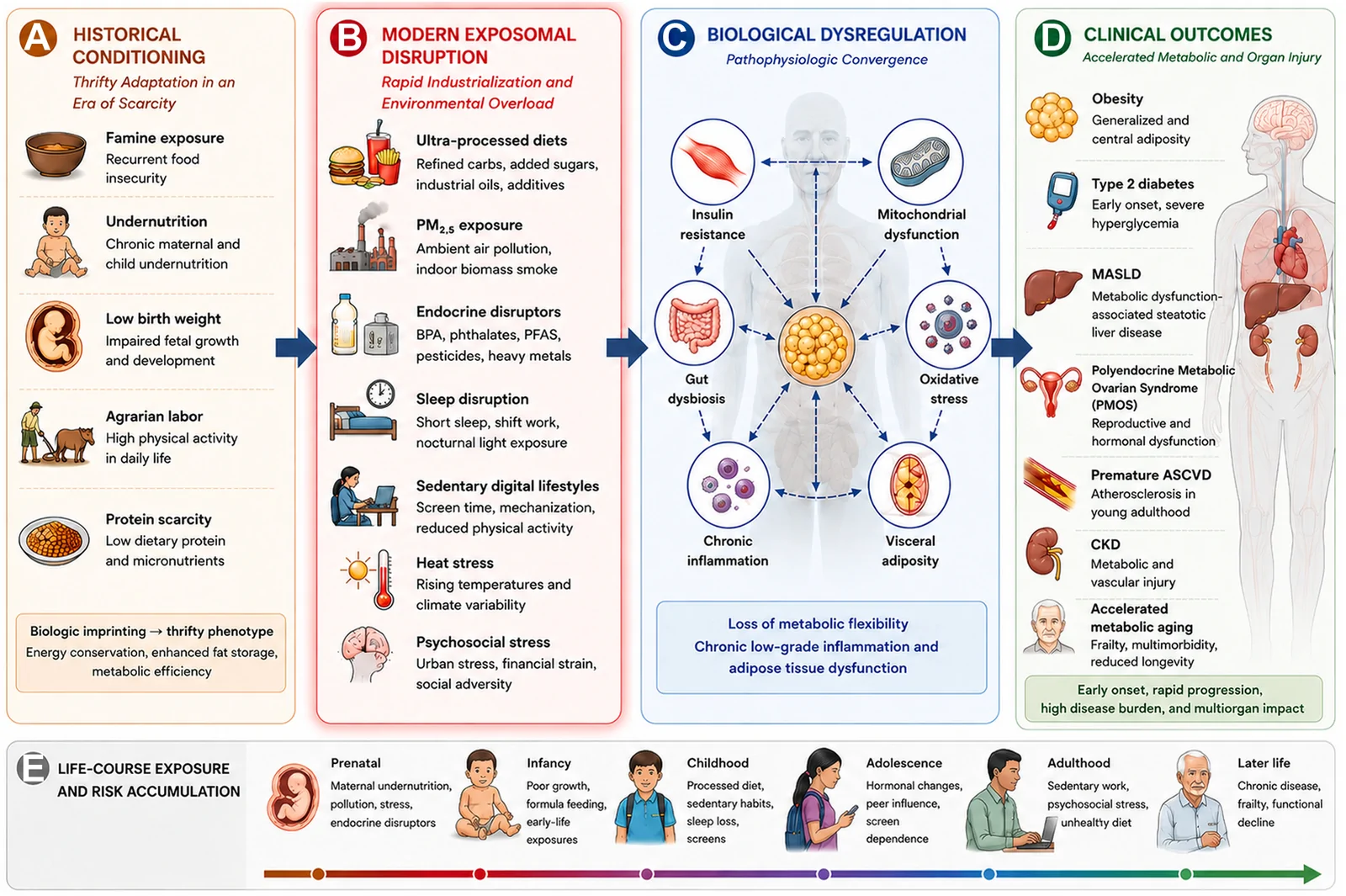
**The South Asian metabolic transition framework.** A five-stage translational framework depicting progression from historical metabolic conditioning and epigenetic priming (A), through modern exposomal disruption (B) and biological dysregulation (C), to early multisystem cardiometabolic disease (D), with life-course accumulation of risk from prenatal life to ageing (E).

The missing link may be the exposome, representing non-conventional exposures across the life-course. Atmospheric air pollution may be among South Asia's most consequential metabolic exposures. Chronic PM_2·5_ levels across the region frequently exceed WHO thresholds and are increasingly linked not only to cardiovascular and pulmonary diseases, but also to insulin resistance, oxidative stress, mitochondrial dysfunction, endothelial injury, and adipocyte dysregulation. Further, EDCs, including bisphenols, phthalates, and per- and polyfluoroalkyl substances, are also widespread and may disrupt adipogenesis, thyroid signalling, appetite regulation, and insulin sensitivity, contributing to obesity, T2D, and broader cardiometabolic disease.^6^ Moreover, circadian disruption, sleep deprivation, irregular eating patterns, shift work, psychosocial stress, and increasing consumption of ultra-processed diets may interact and amplify these effects. Sleep loss promotes insulin resistance and neuroendocrine dysregulation, while dietary and environmental exposures may alter the gut microbiome and inflammatory homoeostasis. Together, these interacting exposures may sustain chronic low-grade inflammation, adipose tissue dysfunction, and vascular injury, providing a plausible pathway to earlier MASLD, T2D, and ASCVD in South Asia.^2^^,^^5^

Thus, South Asia's cardiometabolic epidemic may reflect the cumulative biological consequences of a rapidly changing exposome, superimposed on a population with distinctive metabolic susceptibility. Importantly and we emphasise this point strongly—prevention must move beyond individual lifestyle modification to address the upstream environmental determinants of metabolic health. A new paradigm of precision prevention is therefore needed, centred on four priorities: systematic exposome surveillance incorporating environmental, food, sleep, and climate exposures; early-life and prenatal interventions to reduce metabolic vulnerability from the outset; environmental and food-system policies that complement, rather than rely solely on, individual behavioural interventions; and longitudinal research linking exposomic exposures to cardiometabolic outcomes in South Asian populations. Together, these approaches could shift prevention upstream and may be essential to altering the trajectory of premature cardiometabolic disease across South Asia.

## Contributors

YS, AM: Conceptualisation, Supervision, validation, writing—original draft, visualisation, project administration, and writing–review & editing. All authors approved the final manuscript and were responsible for the decision to submit.

## Declaration of interests

The authors have no relevant interests to disclose.
